# Venetoclax and Bortezomib in Relapsed/Refractory Early T-Cell Precursor Acute Lymphoblastic Leukemia

**DOI:** 10.1200/PO.19.00172

**Published:** 2019-09-20

**Authors:** Roberta La Starza, Benedetta Cambò, Antonio Pierini, Beat Bornhauser, Anna Montanaro, Jean-Pierre Bourquin, Cristina Mecucci, Giovanni Roti

**Affiliations:** ^1^Universitá degli Studi di Perugia, Perugia, Italy; ^2^University of Parma, Parma, Italy; ^3^University Children’s Hospital Zurich, Zurich, Switzerland

## INTRODUCTION

Although in the past three decades we welcomed the advent of targeted therapies in several cancers, nelarabine, a purine nucleoside antimetabolite, remains the most recently approved drug to treat relapsed/refractory (R/R) T-cell acute lymphoblastic leukemia (T-ALL).^1,2^ Patients with R/R T-ALL experience a dramatic outcome, a situation that is more discouraging for the high-risk early T-cell precursor (ETP) ALL subtype.^3^ Historically, ETP ALL is associated with a worse prognosis in children and young adults compared with other T-ALL subtypes because of early resistance to chemotherapy.^4-6^ Although recent data suggest that allogeneic stem-cell transplantation in the first complete remission^7^ may overcome the poor prognosis associated with ETP ALL, novel biologic-based therapies are needed to improve the outcome in these patients. A strategy to overcome the current therapeutic limitations in rare leukemias may involve the development of functional precision medicine approaches on the basis of information by drug response profiling (DRP) of leukemia cells.^8^

## CASE REPORT

**FIG 1.**
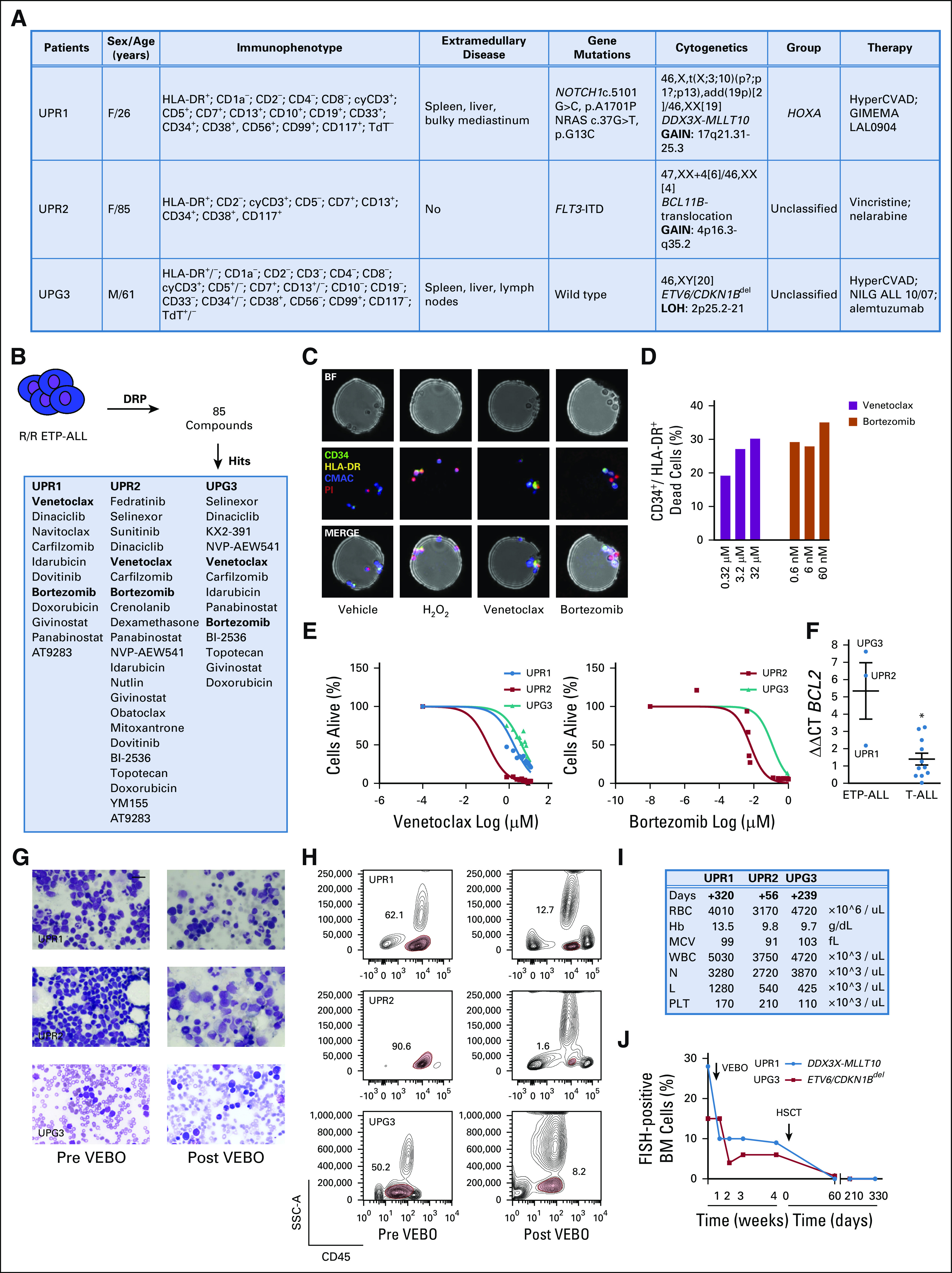
(A) Demographic, clinical, and genomic characteristics of the patients at baseline. Cytogenetic studies include karyotyping, fluorescence in situ hybridization (FISH), and single-nucleotide polymorphism array (question mark indicates undefined chromosome band). Sanger sequencing: *NOTCH1*, *FBXW7*, *PTEN*, *FLT3*, *N/K/H/RAS*, and *BRAF* hot-spot mutations. Immunophenotyping: +/− indicates 40% to 55% positive bone marrow (BM). (B) Identification of active molecules in patients with early T-cell precursor (ETP) acute lymphoblastic leukemia (ALL). ETP leukemia cells were profiled ex vivo with 85 small molecules. Dose-response curves were generated as previously published using five-point drug dilutions in duplicate.^8^ A ranked list of top hits was generated according to the criteria, Emax (ie, the % viability at the maximal drug concentration used) of less than 15% and significant (*P* < .05) difference of half maximal inhibitory concentration (IC50) value for each drug compared with the median IC50 for each particular drug in the reference data set.^8^ In bold: venetoclax and bortezomib (VEBO). (C) Effect of 24 hours of the vehicle (dimethyl sulfoxide), 10 mM hydrogen peroxide (H_2_O_2_), venetoclax, and bortezomib on cellular viability as measured by a functional profiling, on the basis of laboratory-on-a-chip technology, which allows testing the response of live tumor cells exposed to anticancer drugs in an automated process developed by CellPly. ETP ALL cells (eg, patient 2 [UPR2]) were labeled with a 7-amino-4-chloromethylcoumarin (CMAC) dye (blue), an anti–human antigen leukocyte (HLA-DR; green), and an anti-CD34 antibody (yellow) for leukemia cell identification. High-content time-lapsed imaging was performed for 24 hours, every 12 hours, on cells in 480 microwells (75 μm diameter) at a 1 px-1 μm resolution at each time point/condition tested. Cell death was quantified by imaging HLA-DR^+/^CD34^+^ cells positive to the propidium iodide (PI) staining (red). Results are expressed as the percentage of PI^+^/HLA-DR^+/^CD34^+^ positive cells relative to the negative population. (D) Results of C are depicted in the histograms. (E) Effect of venetoclax or bortezomib on cell viability after 72 hours of treatments in available ETP ALL cells as assesses by an ATP-based luminescence viability assay. Error bars denote ± standard deviation (SD) of two replicates. (F) Expression of *BCL2* in ETP ALL and non-ETP ALL (11 cases) as assessed by quantitative real-time polymerase chain reaction. Error bars indicate the mean ± SD of three replicates. Data were analyzed using the ΔΔCT method. (G) BM cytology at relapse (pre VEBO) and after completion of treatment with VEBO (post VEBO; May-Grünwald Giemsa staining at 63×). The marrow contains 50% (patient 1 [UPR1]), 90% (patient 2 [UPR2]), and 28% (patient 3 [UPG3]) of lymphoid blasts before treatment, which decreased rapidly after VEBO. A 500-cell count was performed based on examination of multiple fields to assess hematologic remission (complete remission indicates a blast percentage < 5% in the BM). (H) Antileukemia effect of VEBO treatment assessed by flow cytometry. CD45^+^ staining of primary ETP ALL cells at relapse and after one cycle of VEBO. A minimum of 20,000 events was collected for each condition. (I) Percentage of *DDX3X-MLLT10* (UPR1) and *ETV6/CDKN1B*^del^ (UPG3) FISH-positive BM cells during VEBO treatment and after an allogeneic hematopoietic stem-cell transplantation. The analysis was performed on 500 to 1,000 interphase nuclei for each experiment; the cutoff for *DDX3X-MLLT10* fusion was 2% and for *ETV6/CDKN1B*^del^ was 3%. UPR2 discontinued her BM follow-up after VEBO and retired to a hospice. (J) CBC count on the day of the last hematologic work-up. Days indicate the time elapsed from the last treatment (UPR1 and UPG3 from hematopoietic stem cell transplantation [HSCT], UPR2 from VEBO). BF, bright field; del, deletion; DRP, drug response profiling; Hb, hemoglobin; hyperCVAD, hyperfractionated cyclophosphamide, vincristine, doxorubicin, dexamethasone, and high-dose methotrexate and cytarabine; L, lymphocytes; MCV; mean corpuscular volume; N, neutrophils; NILG, Northern Italy Leukemia Group; PLT, platelets; R/R, relapsed/refractory; SSC-A, side scatter.

Here we report our experience with a salvage treatment regimen that we designed on the basis of recurrent DRP patterns. We used a selection of 85 drugs in three patients with R/R ETP/near-ETP ALL after several lines of chemotherapy (Fig 1A).

### Patient 1

A female patient (UPR1) presented at the hematology and bone marrow transplantation unit of the University of Parma at the age of 26 years with severe bone pain, fatigue, and dry cough unresponsive to over-the-counter analgesics. A WBC count revealed leukocytosis (51,430/cmm), anemia (Hb 9.9 g/dL), and thrombocytopenia (platelets 95,000/cmm). A chest computed tomography scan coupled with an ^18^F-labeled fluorodeoxyglucose–positron emission tomography scan identified a metabolically active lymphadenomegaly in the mediastinum. Flow cytometric analysis identified a bone marrow (BM) blast population (Fig 1A) that was negative for terminal deoxynucleotidyl transferase, CD2, CD4, CD8, and CD1a and positive for human leukocyte antigen (HLA-DR), CD38, CD117, CD33, CD34, CD56, CD99, cyCD3, CD5, CD7, CD10, CD19, and CD13.

Because of a T-ALL immature phenotype^9,10^ and a myeloid interface, and despite CD5 expression, this patient was diagnosed with near-ETP ALL.^11^ Molecular cytogenetic studies showed the presence of *DDX3X-MLLT10* fusion gene derived from a three-way t(X;3;10)(p11;?;p13) translocation. Sanger sequencing detected *NOTCH1* and *NRAS* gain-of-function mutations (Fig 1A). The patient was treated with induction chemotherapy per the HyperCVAD protocol^12,13^ without response and reinduced per the GIMEMA LAL0904 regimen (ClinicalTrials.gov identifier: NCT00458848). A repeated CT scan and marrow examination 2 weeks after salvage chemotherapy showed persistent bulky disease and BM infiltration (47% of leukemic blasts).

### Patient 2

A female patient (UPR2) presented at the hematology and BMT unit of the University of Parma at the age of 78 years with relapsed ETP ALL diagnosed 1 year before (Fig 1A). A WBC count showed leucopenia (1,380/cmm) with severe neutropenia, anemia (Hb 10 g/dL), and normal platelets (257,000/cmm). Molecular cytogenetic studies revealed an abnormal karyotype characterized by an isolated numerical abnormality (ie, 47,XX,+4[6]/46XX[4]), a rearrangement of *BCL11B*, and an internal tandem duplication of *FLT3* (Fig 1A). Because of advanced age and comorbidities, the patient was treated with prednisone 0.5 mg/kg and vincristine 1.4 mg/m^2^ (days 1, 8, 15, and 22), without achieving hematologic remission. A cycle of nelarabine therapy at the reduced schedule of 750 mg/m^2^ on days 1, 3, and 5 was administered, which led to a hematologic improvement for 1 year, when she presented at our hospital with neutropenia (neutrophils 840/cmm) and anemia (Hb 10 g/dL), with a re-expansion of the leukemic clone.

### Patient 3

A male patient (UPG3) presented at the hematology unit of the University Hospital of Perugia with leucopenia (WBC 2,200/cmm), anemia (Hb 11 g/dL), and a moderate thrombocytopenia (platelets 115,000/cmm). Morphologic and flow cytometric analysis of BM cells (Fig 1A) showed a diffuse infiltration of lymphoid blasts negative for CD3, CD2, CD1a, CD8, CD4, CD33, CD117, CD56, and CD10 and positive for CD7, CD38, CD99, cCD3, deoxynucleotidyl transferase, CD34, CD13, CD5 (45%), and HLA-DR, consistent with a diagnosis of ETP ALL.^10^ Cytogenetics showed a normal diploid karyotype. Fluorescence in situ hybridization uncovered a cryptic deletion of the short arm of chromosome 12 involving *ETV6* and *CDKN1B* (Fig 1A). A CT/positron emission tomography scan showed metabolically active cells in the liver, spleen, and lymph nodes. The patient was treated with induction chemotherapy per the HyperCVAD protocol,^12,13^ which led to complete hematologic remission. Two months after the second HyperCVAD cycle, the disease relapsed and the patient was treated with chemotherapy per the Northern Italy Leukemia Group ALL 10/07 trial.^14^ A repeated marrow examination after 2 months showed persistent ETP ALL, and the patient was further treated with an anti-CD52 monoclonal antibody^12^ for a cumulative dose of 100 mg, without benefit.

Because these cases were refractory to repeated standard induction therapy (UPR1, UPG3), or to salvage regimen (UPR2), we decided to support functional precision medicine approaches to repurpose available therapeutic agents. Scoring the response in relation to data for T-ALL on a DRP platform,^8^ the B-cell lymphoma 2 (BCL2) inhibitor BH-3 mimetic venetoclax (ABT-199) and the proteasome inhibitor bortezomib ranked in all three cases among the most active drugs (Fig 1B). Next, we validated the activity of venetoclax and bortezomib using an open microwell microfluidic platform developed by CellPly^15^ (Bologna, Italy; Figs 1C and 1D) and an ATP-based cellular viability assay (Fig 1E) on ETP cells that, consistent with data reported in the literature,^16,17^ express a high level of *BCL2* (Fig 1AF).

On the basis of these findings, individualized treatment with venetoclax and bortezomib (VEBO) was then administered on an outpatient basis, sequentially or in combination, as off-label agents after approval by the relevant institutional review board. All patients received a cycle with venetoclax (800 mg per day × 28 days) by mouth and bortezomib (1.3 mg/m^2^ twice a week × 2 [UPR1, UPR2] or × 4 [UPG3]), with no evidence of major toxicities. UPR1 and UPR2 received antiviral prophylaxis with acyclovir, whereas UPG3 received antimycotic treatment with micafungin. Patients obtained a hematologic complete (UPR2) or partial remission (UPR1 and UPG3) assessed a month after VEBO initial treatment quantified by morphology, flow cytometry analysis, and fluorescence in situ hybridization on bone marrow cells (Figs 1G and 1H). Age-eligible patients (UPR1 and UPG3) underwent an allogeneic stem-cell transplantation, achieving a stable cytogenetic remission (follow-up > 8 months; Fig 1J**)**. All patients are off therapy and alive, with a progressive hematologic recovery (Fig 1I).

## CONCLUSION

Although current chemotherapy regimens result in complete remission in 80% of adults with T-ALL, patients who do not achieve a complete remission or who have a primary resistant disease, including patients with ETP ALL, experience a dramatic poor prognosis.^3,18^ To this end, several groups seek to identify molecularly informed actionable targets^19^ or to develop therapeutic strategies to overcome chemotherapy resistance in T-ALL.^20^ One approach is to develop small molecules on the basis of the identification of tumor dependencies conferred by specific genotypes. This is, for example, the case of γ-secretase inhibitors in *NOTCH1*-mutated T-ALL.^21^

An alternative option is to develop individualized approaches on the basis of pattern of responses to small molecule inhibitors.^22^ A first example was the development of a DRP in primary acute myeloid leukemia samples. The ex vivo testing of 187 drugs in 28 consecutive acute myeloid leukemia cases^22^ led to the identification of five major taxonomic drug-response subtypes with distinct genomic features. Importantly, therapies on the basis of DRP resulted in several clinical responses.^22^ More recently, Frismantas et al^8^ extended this idea and reported a proof-of-concept study by testing 60 drugs in 68 ALL cases. Selected single compounds and combinations were validated in xenograft models. This approach provided compelling evidence that differences in drug response can be pattern clustered in patient groups of interest and reveals patient-to-patient variation.^8^ For example, the authors identified a subset of patients with T-ALL without *ABL1* mutations or fusions responsive to dasatinib inhibition, suggesting an indirect mechanism of sensitivity to dasatinib-based therapy.^8^

Recently, two research groups independently reported that ETP ALL expresses high levels of BCL2 proteins compared with non-ETP ALL cases, resulting in enhanced susceptibility to Bcl2 suppression with BH-3 mimetics, such as ABT-199.^16,23^ Venetoclax as a single agent has been shown to alter proliferation in human T-ALL cell lines and in primary samples, particularly those carrying an ETP phenotype.^16,23^ However, although venetoclax antitumor activity is promising, the rapid emergence of resistance may limit the use of this drug as a single agent.^24,25^ Thus, the downregulation of the antiapoptotic proteins BCL-XL or myeloid cell leukemia-1, which are not targeted by venetoclax, is a rational strategy. In support of this hypothesis, recent work demonstrated that bortezomib, a proteasome inhibitor, induces the BH3-only protein NOXA, which can then downregulate, among other Bcl-2 protein family members, myeloid cell leukemia-1.^17,26^ However, VEBO may go beyond R/R ETP ALL cases. Interestingly Li et al^27^ demonstrated that venetoclax acts synergistically with the MCL1-specific inhibitor S63845 in a broad panel of T-ALL cell lines and in zebrafish embryos undergoing transplantation with T-ALL cells. Moreover, venetoclax showed clinical activity in patients with T-ALL both in combination with chemotherapy^28^ or decitabine,^29^ suggesting that it may be safely included in different therapeutic regimens.

In addition, bortezomib has recently emerged as a potential modulator of the oncogenic T-ALL driver *NOTCH1*. In fact, preclinical studies showed that bortezomib suppresses the expression of Notch and its target genes (*HES1*, *GATA3*, and *RUNX3*) and synergizes with dexamethasone, leading to a near-complete remission of T-ALL xenografted tumors in vivo,^30^ supporting the rationale of several ongoing clinical trials (eg, ClinicalTrials.gov identifiers: NCT02518750 and NCT03643276).

Our results illustrate the potential of functional drug profiling with the identification of an active regimen with less-toxic bioactive drugs for patients with highly resistant R/R T-ALL, and the basis for exploration of this principle for other leukemia subtypes. In conclusion, VEBO represents an effective and well-tolerated chemotherapy-free strategy for R/R T-ALL, including ETP especially, as a bridge to transplantation in fit patients.
